# Percutaneous endoscopic lumbar discectomy combined with platelet-rich plasma injection for lumbar disc herniation: analysis of clinical and imaging outcomes

**DOI:** 10.1186/s12891-024-07444-8

**Published:** 2024-04-24

**Authors:** Tusheng Li, Wei Du, Zhili Ding, Jiang Liu, Yu Ding

**Affiliations:** Orthopedics of TCM Senior Dept, The Sixth Medical Center of PLA General Hospital, Beijing, People’s Republic of China

**Keywords:** Lumbar disc herniation, Platelet-rich plasma, Percutaneous endoscopic lumbar discectomy, Disc degeneration, Repair

## Abstract

**Objective:**

To evaluate the clinical efficacy and imaging outcomes of percutaneous endoscopic lumbar discectomy (PELD) combined with platelet-rich plasma (PRP) for the treatment of lumbar disc herniation (LDH).

**Methods:**

A total of 155 patients with LDH between January 2020 and June 2022 were retrospective analyzed, of which 75 underwent PELD with PRP and 80 underwent PELD only. Clinical functional scores and imaging data were compared. Clinical functional scores included visual analog scale of leg pain (VAS-LP) and back pain (VAS-BP), Japanese Orthopedic Association score (JOA), Oswestry Disability Index (ODI) and modified MacNab criteria. Imaging data included disc height index (DHI), spinal cross-sectional area (SCSA), disc protrusion size (DPZ), and ratio value of disc grey scales (RVG).

**Results:**

Both groups showed clinical improvement, and VAS-LP, VAS-BP, JOA and ODI were significantly improved in the PRP group compared with the control group at 3, 6 and 12 months postoperatively (*P* < 0.05). At the last follow-up, the differences in SCSA, DPZ and RVG between the two groups were statistically significant (*P* < 0.05), with the PRP group being superior to the control group. The excellent and good rates of the modified Macnab criteria in the PRP group and control group were 93.3% and 90%, respectively, with no statistically significant difference (*P* > 0.05). No serious complications occurred during the follow-up period.

**Conclusion:**

PELD combined with PRP is a safe and effective method for treating patients with LDH. PRP injection was beneficial for delaying disc degeneration and promoting disc remodeling.

## Introduction

Lumbar disc herniation (LDH) is a common spinal disease in clinical practice, showing a developing trend of low age and high incidence [1]. For patients with mild symptoms, conservative treatment can alleviate the symptoms, but surgical intervention is still required for patients who do not respond to conservative treatment [2]. Traditional open surgery is a classic procedure for treating LDH, but it requires a large resection of vertebral lamina and facet joints, and extensive stripping of paravertebral muscles, which can lead to complications such as lumbar instability and refractory low back pain [3, 4]. In recent years, minimally invasive spinal endoscopic techniques have attracted increasing attention from scholars worldwide, and are expected to become a superior alternative for the treatment of lumbar degenerative diseases [2, 5]. Currently, percutaneous endoscopic lumbar discectomy (PELD) is an effective spinal endoscopic technique for the treatment of LDH, with advantages such as small trauma, quick recovery, and short hospital stay [6].

Endoscopic discectomy is a widely recognized treatment for LDH, which requires removal of the protruding nucleus pulposus and annulus fibrosus to decompress the nerve root. Appropriate removal of intervertebral discs can reduce patients’ pain symptoms, but aggressive discectomy may lead to further disc degeneration, meanwhile, the defective annulus fibrosus increases the risk of LDH recurrence [7, 8]. Therefore, enhancing intervertebral disc remodeling after discectomy is a critical factor for improving surgical prognosis. In this context, advances in regenerative medicine have spurred the development of experiments aimed at restoring and reconstructing healthy discs, notably growth factor therapy, cell therapy and gene therapy [9]. Platelet-rich plasma (PRP) is a concentrated platelet product obtained through the centrifugation of whole blood, rich in various growth factors, showing promise in repairing intervertebral disc tissues in in vitro and animal experiments [10]. Studies have shown that PRP can promote tissue repair and healing effects by regulating the binding of growth factors to extracellular receptors on target cells and synergistically mediating the expression of intracellular signal transduction pathways [11, 12]. Additionally, PRP exhibits anti-inflammatory potential by downregulating the expression of inflammatory factors [10]. At present, spinal endoscopy combined with PRP for the treatment of LDH is attracting increasing clinical attention. In previous studies, Zhang [1] and Jiang [13] reported that endoscopic discectomy combined with PRP for LDH could achieve satisfactory clinical outcomes. However, the evidence for PRP to delay disc degeneration and promote disc remodeling is still limited, especially in terms of imaging. In this study, the clinical and imaging results of PELD combined with PRP for LDH were retrospectively analyzed, aiming to determine whether PRP can provide better clinical outcomes and enhance intervertebral disc repair.

## Materials and methods

### Study design and patients

This was a single-center retrospective cohort study conducted with the approval of the Ethics Committee of the Sixth Medical Center of the PLA General Hospital (No. HZKY-PJ-2023-4), and all patients provided written informed consent before treatment. The study was conducted in accordance with the principles of the Declaration of Helsinki.

The inclusion criteria were as follows: (1) single-level disc herniation, with both symptoms of low back pain and leg pain; (2) age between 20 and 65 years; (3) failed conservative treatment after 8 weeks; (4) platelet count > 150 × 10^9^/L; (5) stable vital signs. The exclusion criteria were as follows: (1) LDH with calcification of the posterior longitudinal ligament; (2) LDH with lumbar instability, infection, tumor, or deformity; (3) cauda equina syndrome; (4) previous lumbar surgery history; (5) pregnancy; (6) incomplete follow-up information.

A total of 155 LDH patients were included in the study between January 2020 and June 2022. The cohort comprised 75 patients who underwent PRP combined with PELD and 80 patients who underwent PELD only. All patients provided written informed consent before treatment.

### PRP preparation

**Fig. 1:**
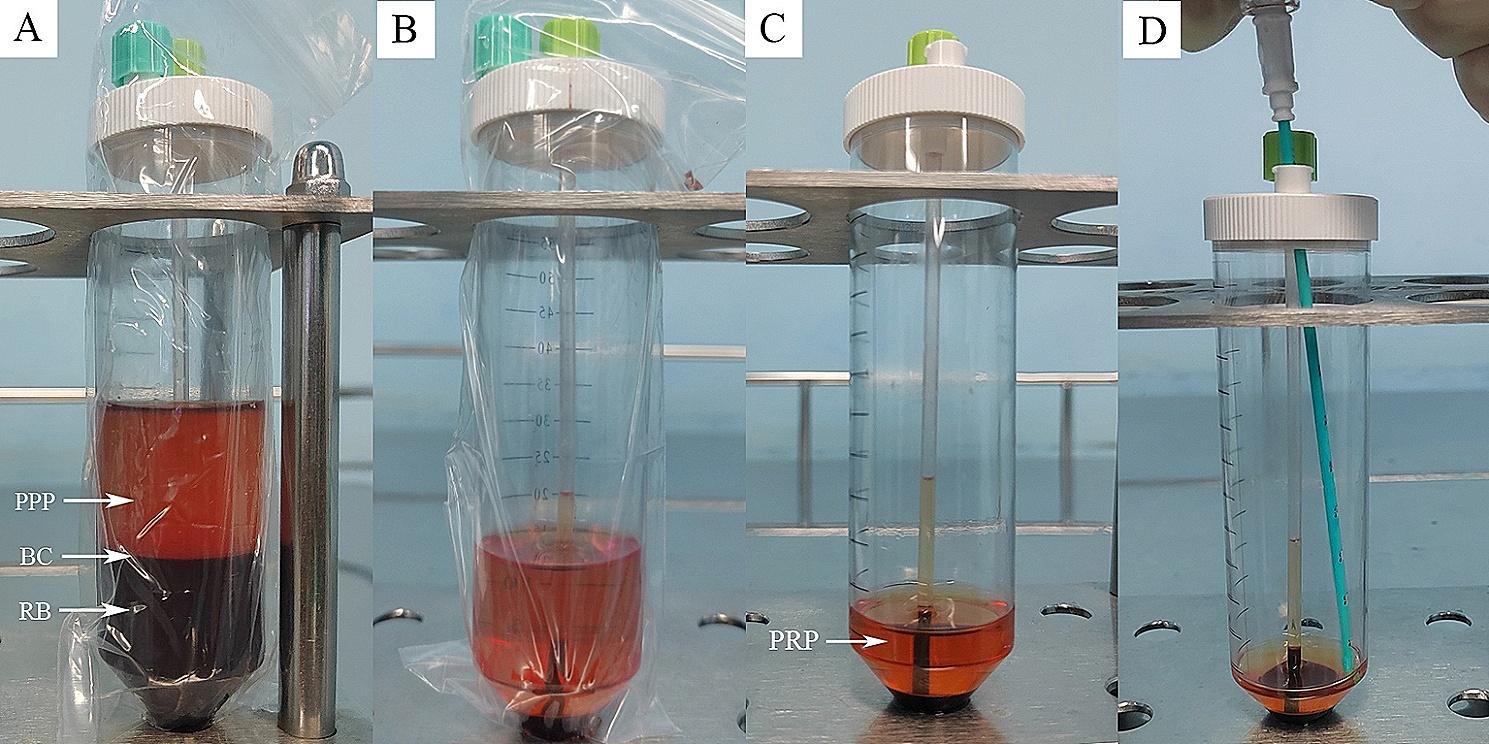
Schematic diagram of PRP preparation. **A** PRP after one-step centrifugation (PPP: platelet-poor plasma layer; BC: buffy coat layer; RB: red blood cell layer). **B** PRP after two-step centrifugation. **C** After aspirating part of the supernatant, the remaining supernatant is the required PRP. **D** Aspiration of PRP into syringe

The PRP was prepared using the sterile WEGO PRP kit (Weigo Medical Polymer Products Co., Ltd., Shandong, China). Firstly, 4 ml of citrate anticoagulant was added to a sterile tube, and 36 ml of fresh venous blood was drawn from the patient’s median cubital vein (1 ml citrate anticoagulant per 9 ml of blood). The whole blood containing the anticoagulant was then subjected to the first centrifugation at 2000 rpm for 10 min and 30 s. After centrifugation, the blood was separated into three layers, with a platelet-poor plasma layer (PPP) at the top, a buffy coat layer (BC) in the middle, and a red blood cell layer (RB) at the bottom. The bottommost layer of red blood cells was aspirated from the tube. Subsequently, a second centrifugation was performed at 2350 rpm for 10 min and 30 s to further enrich the platelets. After centrifugation, the supernatant was removed and the remaining liquid was the desired PRP (Fig. 1). The PRP volume for the injection was approximately 4 ml.

### Surgical procedure

**Fig. 2:**
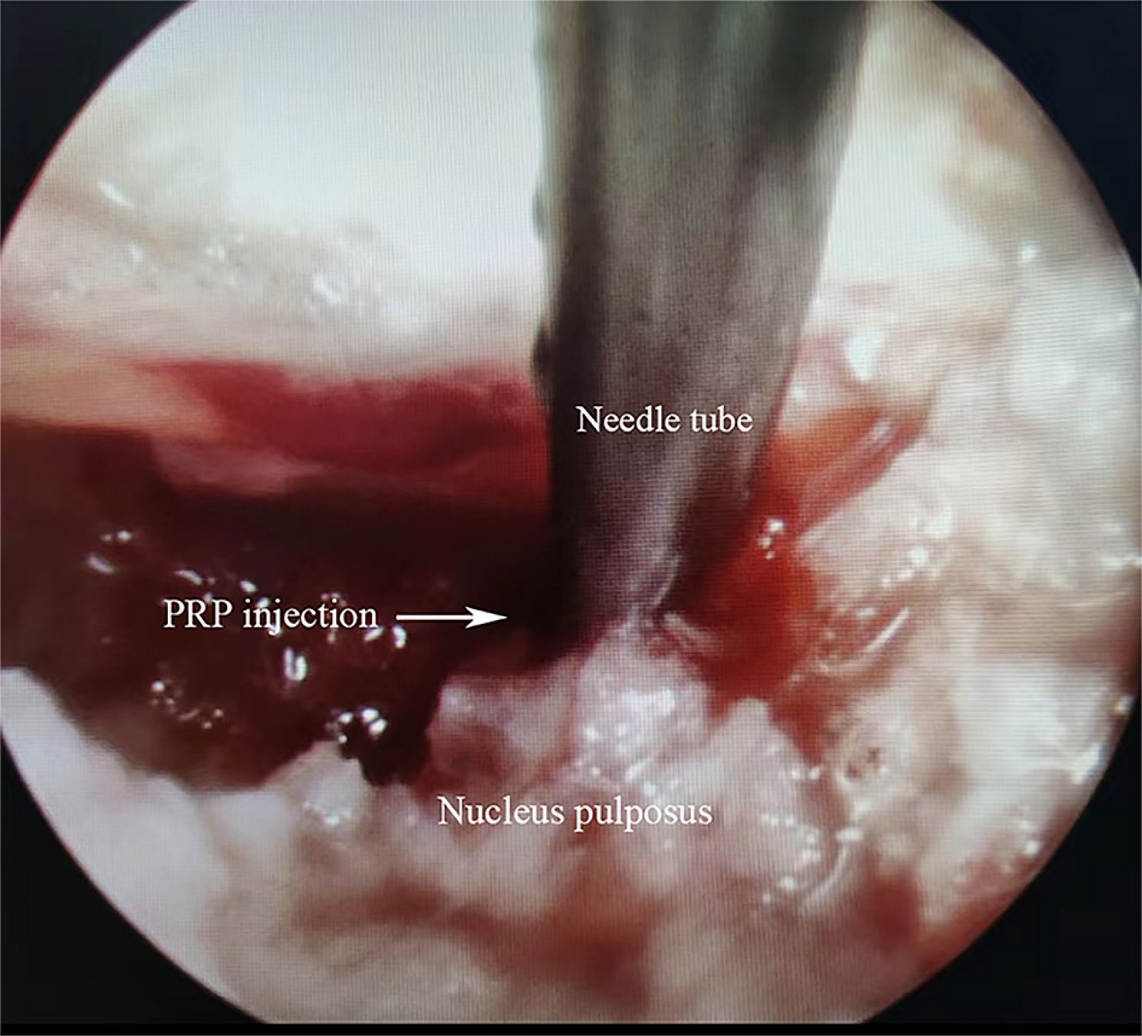
Schematic of PRP injection into the decompressed disc under endoscopy

**Fig. 3:**
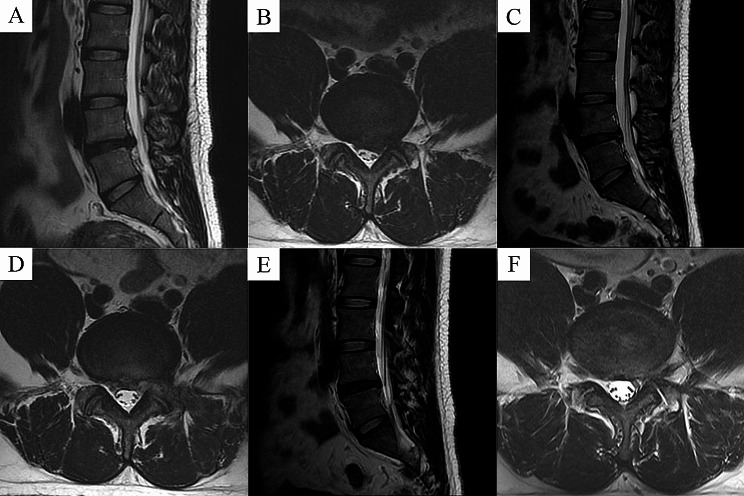
Representative images of patients in the PRP group. **A** and **B** Preoperative MRI showed L4-5disc herniation with nerve root compression. **C** and **D** At 3 months postoperatively, MRI showed removal of the herniated disc, and adequate decompression of the spinal canal. **E** and **F** At 12 months postoperatively, MRI showed further decompression of the spinal canal

All surgeries were performed under local anesthesia with 0.5% lidocaine by the same experienced surgeon using standard techniques. Patients were placed in a prone position, and the surgical segment was confirmed under C-arm fluoroscopy. A puncture needle was inserted, and the tip was verified to be located on the lateral aspect of the superior facet under fluoroscopy. After that, a guidewire was introduced and a 7 mm incision was made along the guide wire. A series of dilators were used to expand the surgical channel, and a working cannula was inserted. Then, the spinal endoscope system was connected, and the endoscope was placed. Soft tissue was cleared using a bipolar radiofrequency knife under endoscopic guidance to create an open surgical field. Intraoperatively, foraminoplasty was performed as needed. The protruding nucleus pulposus and annulus fibrosus were removed under endoscopy, and the nerve root was explored and decompressed. After draining out the irrigation fluid, in the PRP group, the fresh PRP fluid prepared and 0.4 ml of thrombin (1:10) was aspirated using two sterile syringes, respectively. Two syringes and a long puncture needle (16G) were attached to three-way tube. Under endoscopy, the puncture needle was confirmed to be located within the decompressed disc, and then PRP was injected simultaneously with thrombin. Thrombin activated platelets and formed a PRP gel mixture within the disc. Subsequently, gelatine hemostatic sponges were covered with annulus fibrosus notches. In the control group, discectomy was performed without PRP injection. Both groups had their incisions closed without drainage. PRP injection under endoscopy is shown in Fig. 2. Representative case in the PRP group is shown in Fig. 3.

### Outcomes assessment

Demographic characteristics, platelet counts, and the occurrence of complications were collected from all patients. Clinical functional assessments were determined at 3 days postoperatively, and at 3, 6, and 12 months postoperatively through self-assessment questionnaires. Patients’ clinical pain was assessed using Visual Analog Scale of back pain (VAS-BP) and leg pain (VAS-LP), and lumbar functional dysfunction was assessed using Japanese Orthopedic Association score (JOA) and Oswestry Disability Index (ODI). At 12 months postoperatively, patient satisfaction was rated as excellent, good, fair, or poor using the Modified MacNab criteria.

### Radiographic evaluation

Both groups of patients underwent lumbar spine X-rays and magnetic resonance imaging (MRI) examinations preoperatively, and at 3, and12 months postoperatively. Imaging data were collected in DICOM or JPG format and measured using Image Viewer or AnyPacs software installed on workstations. All imaging data were measured three times by three independent assessors, and the averages were recorded.

**Fig. 4:**
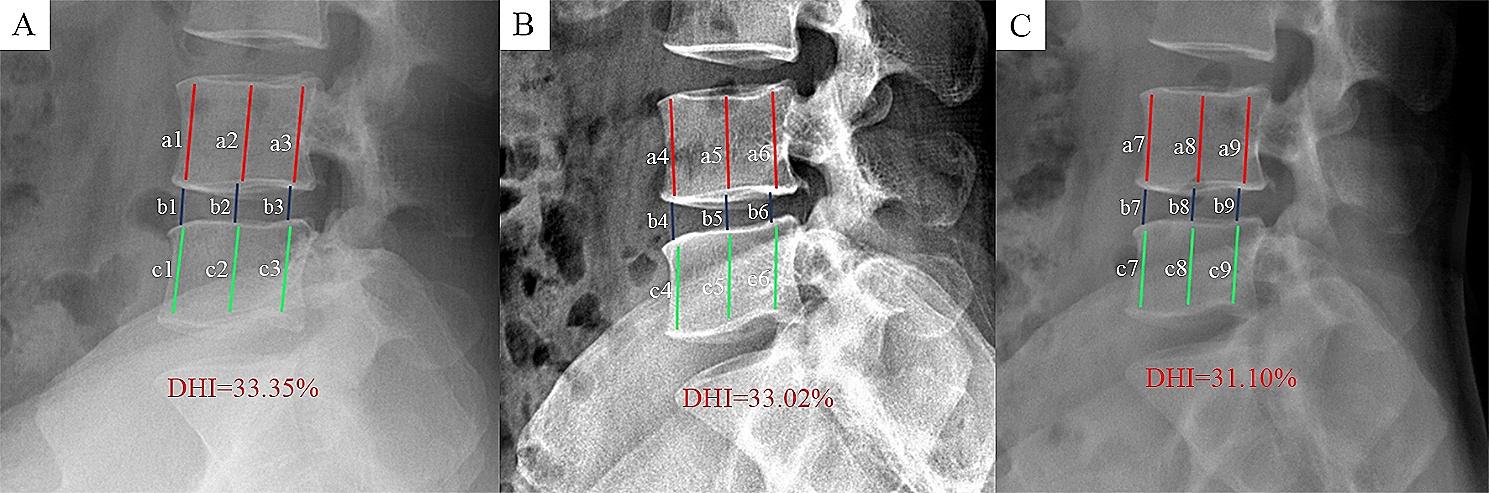
Schematic diagram of DHI measurement, DHI = [2(disc height)] / [(height of the upper vertebrae) + (height of the lower vertebrae)] *100%. Comparison of DHI on lumbar X-ray preoperatively (**A**), 3 months (**B**) and 12 months (**C**) postoperatively

**Fig. 5:**
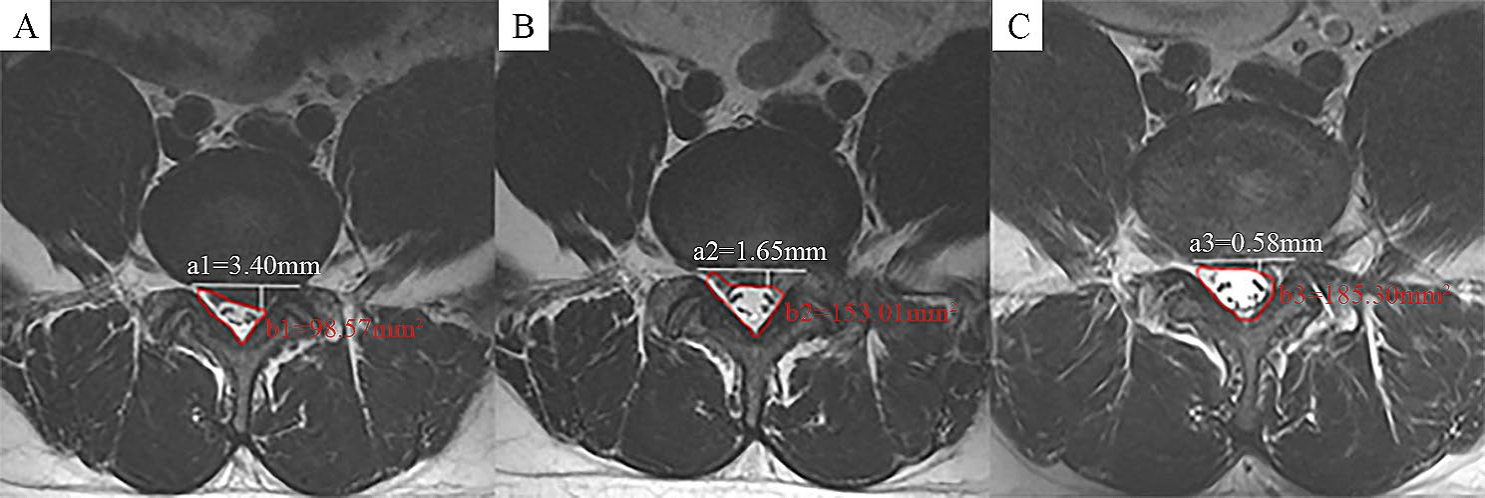
Schematic diagram of SCSA (indicated in red, b) and DPZ (indicated in white, a) measurements. Comparison of SCSA and DPZ on MRI preoperatively (**A**), 3 months (**B**) and 12 months r (**C**) postoperatively

**Fig. 6:**
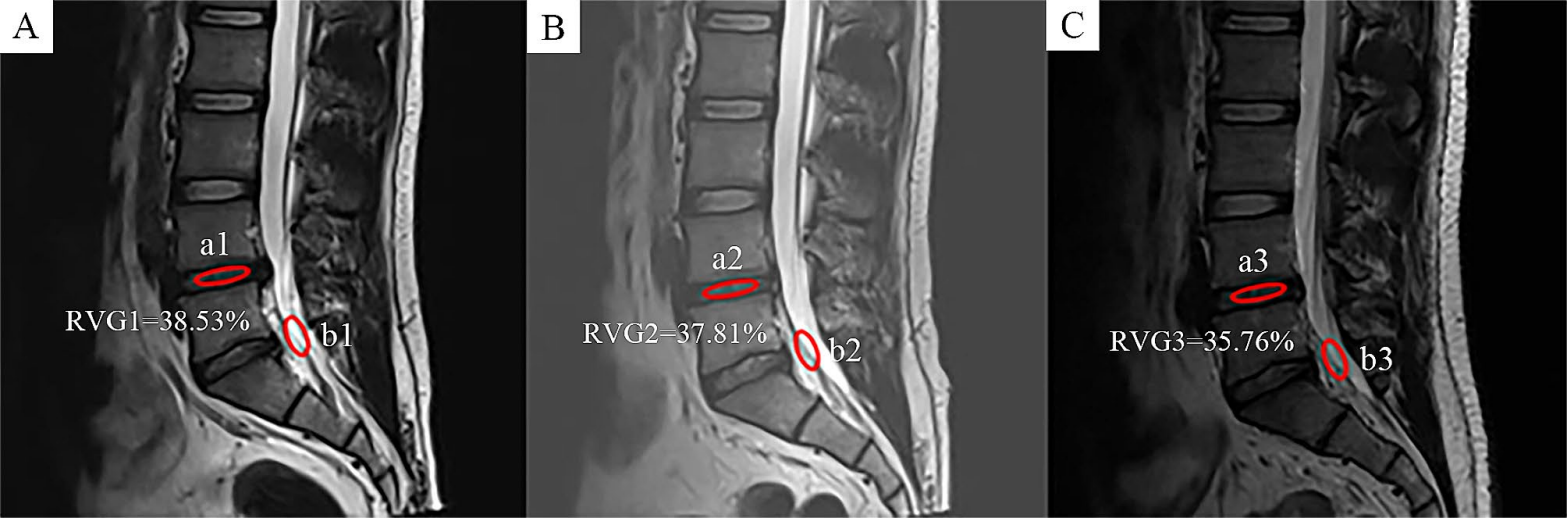
Schematic diagram of RVG measurement. The cerebrospinal fluid grey scale of sacral one segment (b) was selected as a reference, and the grey scale of the responsible segmental disc (a) was measured, RVG = (a / b) * 100%. Comparison of RVG on MRI preoperatively (**A**), 3 months (**B**) and 12 months (**C**) postoperatively

(1) Disc height index (DHI): DHI was used to assess changes in disc height at different follow-up time points, as previously described [9]. The anterior, middle, and posterior heights of the upper and lower vertebral bodies and discs were measured on lateral lumbar spine X-ray. DHI is calculated as the ratio of disc height to the average height of the upper and lower vertebrae, as shown in Fig. 4.

(2) Spinal canal cross-sectional area (SCSA): The cross-sectional area of the spinal canal was measured on the MRI axial position as in previous studies [13]. SCSA was used to evaluate the degree of improvement of the spinal canal at different follow-up time points, as shown in Fig. 5.

(3) Disc protrusion size (DPZ): The DPZ was measured on the MRI axial position by drawing a line at the bottom of the disc and then making a vertical line to the point where the disc is most herniated [13]. DPZ was applied to analyze the size of disc protrusion over time, as shown in Fig. 5.

(4) Ratio value of disc grey scales (RVG): The measurement of RVG was based on the modified Schneiderman method for assessing the water content of disc [9]. The cerebrospinal fluid grey scale of sacral one segment was selected as the baseline reference value, and the grey scale of the responsible segmental disc was measured. RVG = (average grayscale value of the responsible intervertebral disc / average grayscale value of cerebrospinal fluid sacral one segment) * 100%, as shown in Fig. 6.

### Statistical analysis

All statistical analyzes were performed using SPSS version 25 (IBM SPSS Statistics for Windows, Version 25.0. Armonk, NY: IBM Corp.). For normally distributed continuous data, Student’s t-test analysis was conducted and results were expressed as means ± (SD). Comparisons between time points within each group were analyzed using repeated measures analysis of variance. For non-normally distributed data, non-parametric tests were used. Categorical data were presented as frequencies and percentages and compared using the chi-square test. Reliability of independent reviewers was calculated using the intraclass correlation coefficient (ICC), with an ICC greater than 0.75 indicating good agreement. A significance level of *P* < 0.05 indicated a statistically significant difference between the two groups.

## Results

All patients completed 1 year of follow-up. Baseline characteristics showed no significant differences between the two groups (Table 1).

**Table 1:** Comparison of demographics between the two groups (mean ± SD)

Demographics	PRP group (*n* = 75)	Control group (*n* = 80)	*P* value
Age (years)	43.61 ± 11.72	44.25 ± 11.56	0.734
BMI (kg/m^2^)	24.22 ± 2.98	24.41 ± 3.09	0.693
Gender, n (%)			0.492
Male	39 (52.0)	46 (57.5)	
Female	36 (48.0)	34 (42.5)	
Medical history, n (%)			
Hypertension	13 (17.3)	16 (20.0)	0.671
Diabetes	20 (26.7)	27 (33.8)	0.338
Operative segment			0.883
L3-4	10 (13.3)	12 (15.0)	
L4-5	36 (48.0)	40 (50.0)	
L5-S1	29 (38.7)	28 (35.0)	
Herniation type, n (%)			0.931
Protrusion	30 (40.0)	30 (37.5)	
Extrusion	35 (46.7)	38 (47.5)	
Free	10 (13.3)	12 (15.0)	
Herniated disc location, n (%)			0.857
Central	22 (29.3)	22 (27.5)	
Paracentral	32 (42.7)	36 (48.0)	
Foraminal	19 (25.3)	18 (22.5)	
Extreme lateral	2 (2.7)	4 (5.0)	
Disease duration (months)	21.87 ± 7.86	23.54 ± 8.40	0.204
Smoking, n (%)	17 (22.7)	22 (27.5)	0.488
Platelet levels (×10^9^/L)	211.25 ± 28.84	214.30 ± 30.29	0.523

SD, standard deviation; BMI, body mass index; PRP, platelet-rich plasma

### Clinical evaluation

**Fig. 7:**
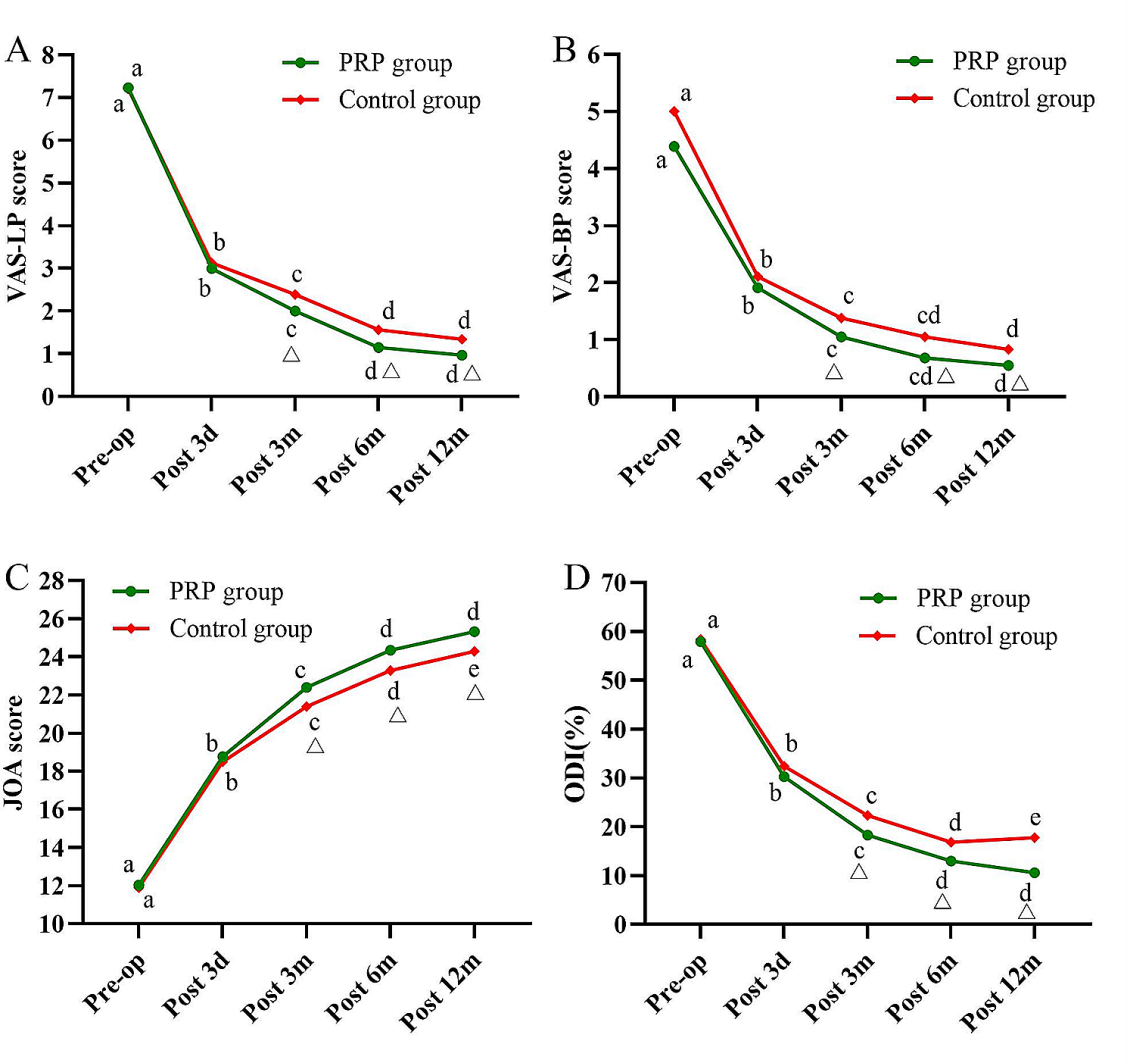
Results of clinical functional scores. **A** Changes in VAS-LP scores over time. **B** Changes in VAS-BP scores over time. **C** Changes in JOA scores over time. **D** Changes in ODI scores over time. VAS, Visual Analog Scale; JOA, Japanese Orthopedic Association; ODI, Oswestry Disability Index. a-e indicate the letter labelling of the time point difference (comparison within the group), if 2 time points have the same letter, there is no significant difference between the 2 time points (*P* > 0.05); otherwise, different letters at 2-time points mean the difference is significant (*P* ≤ 0.05). Δ represents a significant difference between the two groups

The mean VAS-LP scores in the PRP and control groups decreased from 7.23 ± 1.06 and 7.20 ± 1.16 before operation (*P* = 0.855) to 3.00 ± 0.81 and 3.13 ± 0.83 at 3 days postoperatively (*P* = 0.389); 2.00 ± 0.74 and 2.39 ± 0.93 at 3 months postoperatively (*P* = 0.023); 1.15 ± 0.71 and 1.56 ± 1.00 at 6 months postoperatively (*P* = 0.017); and 0.97 ± 0.79 and 1.34 ± 1.02 (*P* = 0.041) at 12 months postoperatively. In addition, the mean VAS-BP scores in the PRP and control groups decreased from 4.93 ± 1.32 and 5.00 ± 1.36 before operation (*P* = 0.697) to 1.91 ± 1.03 and 2.11 ± 1.18 at 3 days postoperatively (*P* = 0.217); 1.05 ± 0.93 and 1.38 ± 1.04 at 3 months postoperatively (*P* = 0.047); 0.68 ± 0.72 and 1.05 ± 0.98 at 6 months postoperatively (*P* = 0.023); and 0.55 ± 0.70 and 0.83 ± 0.84 (*P* = 0.038) at 12 months postoperatively. The PRP group had lower VAS-LP and VAS-BP scores at 3, 6, and 12 months postoperatively compared to the control group, with statistically significant differences (*P* < 0.05). Both groups showed significant improvement in VAS-LP and VAS-BP scores postoperatively compared to preoperatively (*P* < 0.05) (Fig. 7A and B).

The mean JOA scores in the PRP and control groups significantly improved from 12.03 ± 1.79 and 11.89 ± 1.84 before operation (*P* = 0.624) to 18.77 ± 1.82 and 18.50 ± 1.89 at 3 days postoperatively (*P* = 0.380); 22.39 ± 2.50 and 21.40 ± 2.73 at 3 months postoperatively (*P* = 0.045); 24.35 ± 2.06 and 23.28 ± 2.32 at 6 months postoperatively (*P* = 0.015); and 25.33 ± 2.35 and 24.28 ± 2.48 at 12 months postoperatively (*P* = 0.014). The PRP group showed better improvement in JOA scores than the control group at 3, 6, and 12 months postoperatively, and the difference was statistically significant (*P* < 0.05). Postoperative JOA scores improved significantly in both groups compared with preoperative (*P* < 0.05) (Fig. 7C).

The mean ODI in the PRP and control groups decreased from 57.92 ± 12.73 and 58.38 ± 13.19 before operation (*P* = 0.828) to 30.24 ± 9.40 and 32.40 ± 9.47 at 3 days postoperatively (*P* = 0.119); 18.29 ± 8.31 and 22.33 ± 9.00 at 3 months postoperatively (*P* = 0.003); 12.96 ± 7.48 and 16.83 ± 9.31 at 6 months postoperatively (*P* = 0.028); and 10.59 ± 7.41 and 17.75 ± 9.05 at 12 months postoperatively. The PRP group showed better improvement in ODI than the control group at 3, 6, and 12 months postoperatively, and the difference was statistically significant (*P* < 0.05). Both groups showed significant improvement (*P* < 0.05) in postoperative ODI compared with preoperative (*P* < 0.05) (Fig. 7D).

At the last follow-up, according to the modified MacNab criteria, there were 43 cases of excellent, 27 cases of good, 5 cases of fair, and 0 cases of poor in the PRP group, with an excellence and good rate of 93.3%; there were 32 cases of excellent, 40 cases of good, 8 cases of fair, and 0 case of poor in the control group, with an excellence and good rate of 90.0%. There was no statistically significant difference in the comparison of the excellent and good rate between the two groups (*P* = 0.454).

### Image measurement

**Fig. 8:**
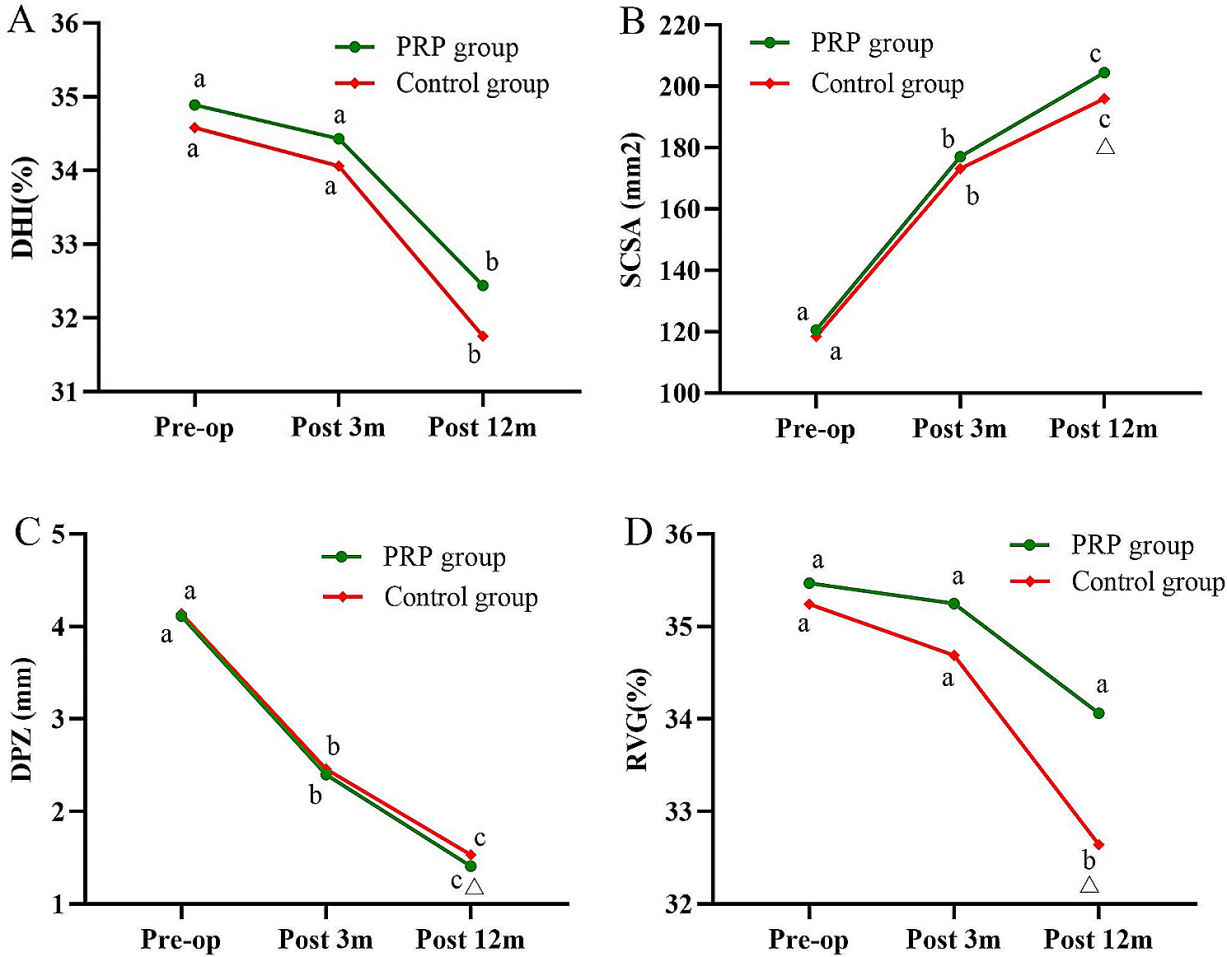
Results of imaging measurement. **A** Changes in DHI during the follow-up. **B** Changes in SCSA during the follow-up. **C** Changes in DPZ during the follow-up; D Changes in RVG during the follow-up. DHI, Disc Height Index; SCSA, spinal cross-sectional area, DPZ, disc protrusion size; RVG, ratio value of disc grey scales. a-c indicate the letter labelling of the time point difference (comparison within the group); if 2 time points have the same letter, there is no significant difference between the 2 time points (*P* > 0.05); otherwise, different letters at 2-time points mean the difference is significant (*P* ≤ 0.05). Δ represents a significant difference between the two groups

In both groups, the DHI of the responsible segment showed a decreasing trend. The mean DHI in the PRP and control groups decreased from (34.89 ± 4.20) % and (34.58 ± 4.07) % before operation (*P* = 0.644) to (34.43 ± 4.06) % and (34.06 ± 3.90) % at 3 months postoperatively (*P* = 0.558); and (32.44 ± 3.96) % and (31.75 ± 3.81) % at 12 months postoperatively (*P* = 0.275), respectively. There was no statistically significant difference in DHI between the two groups (*P* > 0.05) (Fig. 8A).

The mean DCSA in the PRP and control groups significantly improved from (120.59 ± 14.34) mm^2^ and (118.48 ± 15.45) mm^2^ before operation (*P* = 0.380) to (177.05 ± 16.15) mm^2^ and (173.14 ± 17.03) mm^2^ at 3 months postoperatively (*P* = 0.145); and (204.41 ± 16.57) mm^2^ and (195.97 ± 16.50) mm^2^ at 12 months postoperatively (*P* = 0.002), respectively. SCSA improved significantly in the PRP group compared with the control group at 12 months postoperatively (*P* < 0.05) (Fig. 8B).

The mean DPZ in the PRP and control groups decreased from (4.11 ± 0.68) mm and (4.14 ± 0.66) mm before operation (*P* = 0.299) to (2.40 ± 0.35) mm and (2.46 ± 0.37) mm at 3 months postoperatively (*P* = 0.166); and (1.41 ± 0.30) mm and (1.53 ± 0.36) mm at 12 months postoperatively (*P* = 0.042), respectively. At 12 months postoperatively, DPZ improved significantly in the PRP group compared with the control group (*P* < 0.05) (Fig. 8C).

In both groups, the RVG showed a decreasing trend. The mean RVG in the PRP and control groups decreased from (35.47 ± 3.88) % and (35.24 ± 3.82) % before operation (*P* = 0.704) to (35.25 ± 3.84) % and (34.69 ± 3.76) % at 3 months postoperatively (*P* = 0.360); and (34.06 ± 3.77) % and (32.64 ± 3.70) % at 12 months postoperatively (*P* = 0.019), respectively. At 12 months postoperatively, RVG decreased more in the control group than in the PRP group (*P* < 0.05) (Fig. 8D).

In terms of imaging, the PRP group was better than the control group in terms of SCSA improvement and DPZ reduction, indicating that the application of PRP increased endogenous repair of the disc and connected better with the residual annulus fibrosus. The decrease in RVG was smaller in the PRP group than in the control group (*P* < 0.05), suggesting that PRP contributed to delaying intervertebral disc degeneration. In addition, we observed that the ICC for DHI, SCSA, DPZ and RVG were all greater than 0.75 (0.773–0.974), indicating good inter-rater reliability (Table 2).

**Table 2:** Comparison of ICC between independent reviewers

Characteristics	ICC	95%CI
DHI		
Pre-op	0.866	0.769–0.917
3 months	0.867	0.771–0.918
12 months	0.881	0.832–0.915
SCSA		
Pre-op	0.802	0.752–0.846
3 months	0.773	0.717–0.822
12 months	0.856	0.817–0.888
DPZ		
Pre-op	0.865	0.826–0.896
3 months	0.779	0.722–0.827
12 months	0.881	0.848–0.909
RVG		
Pre-op	0.854	0.809–0.889
3 months	0.913	0.885–0.935
12 months	0.950	0.934–0.962

DHI, disc height index; SCSA, spinal cross-sectional area; DPZ, disc protrusion size; RVG, ratio value of disc grey scales; ICC, intraclass correlation coefficient

### Complications

**Fig. 9:**
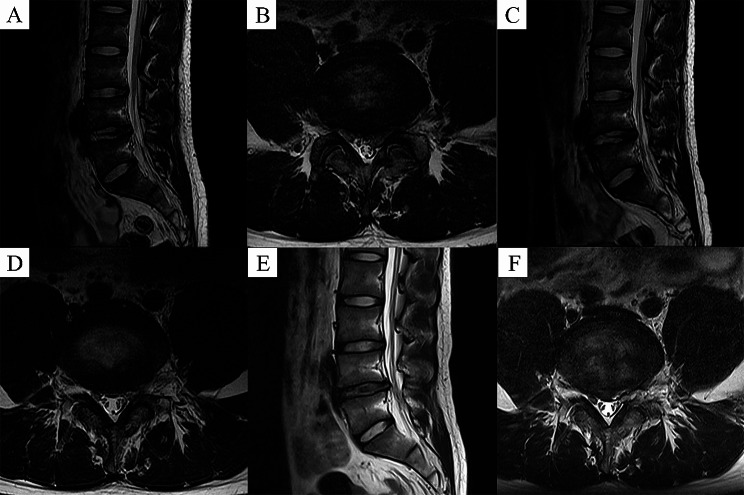
Imaging of recurrent cases in the PRP group. **A** and **B** Preoperative MRI showed L4-5disc herniation with nerve root compression. **C** and **D** At 3 months postoperatively, MRI showed removal of the herniated disc, and adequate decompression of the spinal canal. **E** and **F** At 8 months postoperatively, MRI showed recurrent herniated lumbar disc

During the follow-up period, 8 patients required revision surgery due to recurrence. Of these, 1 patient were in the PRP group (recurrence rate of 1.33%), and 7 patients were in the control group (recurrence rate of 8.75%). All patients were treated with a second endoscopic spinal surgery of the original responsible segment, and had postoperative symptomatic relief. The difference in LDH recurrence rates between the two groups was not statistically significant (*P* = 0.085). No serious complications such as dural tear or disc space infection occurred in any of the patients. Representative case of recurrence in the PRP group is shown in Fig. 9.

## Discussion

The combination of PELD with PRP injection provides better clinical outcomes for LDH patients compared to PELD only. In this study, patients treated with PRP injection demonstrated more significant improvements in postoperative VAS-LP, VAS-BP, JOA, and ODI scores and exhibited advantages in promoting intervertebral disc remodeling and delaying disc degeneration in terms of imaging. None of the patients in this study showed signs of segmental instability, or cauda equina syndrome based on both radiographic and clinical assessments.

LDH is a frequent and debilitating disease that seriously impacts global health and adds to the economic burden [14]. LDH leads to nerve root compression prone to radiating pain in the lower limbs, thereby adequate nerve root decompression is crucial for treatment [13]. However, the surgical decompression process inevitably disrupts the normal structure of the intervertebral disc, which may cause worsened disc degeneration postoperatively. Additionally, local inflammatory reactions at the surgical site due to various factors can also affect patient outcomes [15]. With the development of regenerative medicine, PRP has shown promising potential as a novel and safe biological approach in the treatment of degenerative disc diseases [10]. Surgery in conjunction with biological therapy is emerging as a new concept to achieve both symptom relief and prevention of secondary diseases [9]. PRP activation releases a multitude of growth factors, which have been demonstrated in in vitro and animal experiments to have effects such as downregulating inflammatory factor expression, promoting angiogenesis, inhibiting cell apoptosis, facilitating intervertebral disc cell regeneration, and supporting nerve function recovery [10–12]. In terms of clinical research, Akeda et al. [16] conducted an initial clinical trial in 2011, providing the first evidence that intra-disc application of autologous PRP is an effective treatment for degenerative disc diseases. Since then, numerous scholars have reported the application of PRP in alleviating back pain and neurogenic symptoms, with sustained long-term clinical effects [1, 13, 17]. In our study, patients treated with PRP injection demonstrated a greater advantage in terms of pain relief and neurological function improvement, aligning with the outcomes of previous trials.

The exacerbation of intervertebral disc degeneration after endoscopic discectomy is a critical factor in LDH recurrence and persistent symptoms. The principle behind PRP treatment for degenerative intervertebral discs involves the direct application of high concentrations of growth factors to intervertebral disc cells, thereby stimulating endogenous repair mechanisms and improving function [17]. In our study, we observed that both groups exhibited degenerative changes in RVG in the intervertebral disc after PELD. However, patients receiving PRP injection treatment showed a smaller decrease in RVG at the last follow-up, indicating that PRP application had delayed disc degeneration to some extent. The loss of intervertebral disc height is a natural occurrence following lumbar discectomy. Previous animal experiments have suggested that PRP application can effectively restore intervertebral disc height [18], nevertheless, in our study, we found that both groups showed a decrease in DHI within one year, and there was no significant difference between them. We suggest that this discrepancy may be due to differences in intervertebral disc biomechanics between animal models and the human body [13]. Additionally, whether the continued reduction in DHI will impact patient surgical outcomes needs to be studied further.

PRP injection treatment for LDH not only delays intervertebral disc degeneration but also effectively repairs the defective annulus fibrosus. Over time, we found that patients receiving PRP injection treatment demonstrated an advantage in terms of SCSA improvement and DPZ reduction. This suggests that PRP plays a proactive role in annulus fibrosus repair and contributes to the reconstruction of intervertebral disc tissue [13, 19]. Platelets and fibrin in PRP can adhere to annulus fibrosus cells, which contributes to effectively seal fissures in the annulus fibrosus [12, 17]. Fibrin is easily malleable and possesses ideal characteristics for intervertebral disc cells, making it a potential adhesive for repairing the annulus fibrosus [20]. In addition, PRP can promote the proliferation and differentiation of chondrocytes in the annulus fibrosus, transforming annulus fibrosus tissue into cartilaginous tissue [21]. This can effectively reduce the formation of scar tissue within the intervertebral disc while increasing the formation of extracellular matrix in vitro, contributing to disc repair [21, 22]. Annulus fibrosus repair creates a physical barrier between the disc contents and blood, helping reduce the formation of immune inflammatory reactions and minimizing postoperative pain symptoms [20, 23].

The safety of PRP injection was also a focal point of this study. PRP is derived from the patient’s own blood, with no risk of disease transmission, infection, or allergic reactions [24]. It has been reported that PRP possesses antimicrobial properties, which can effectively reduce the risk of postoperative infections [25]. Furthermore, previous studies have indicated that certain white blood cells within PRP, such as neutrophils, monocytes, and lymphocytes, play important roles in the body’s inflammatory response and infection control [12]. During the follow-up period, the PRP group did not experience complications such as intervertebral disc infection or worsening of nerve root symptoms.

There are some limitations in the current study. Firstly, it is a single-center retrospective study, which cannot achieve double-blinding or random group allocation, and was prone to selection error in patient enrolment. Secondly, the limited number of cases and short follow-up duration may influence the study results. Thirdly, although all imaging results were averaged over 3 measurements by 3 independent reviewers, measurement error can still exist.

## Conclusion

Our study showed that PELD combined with PRP is a safe and effective method for treating patients with LDH. PRP injection was beneficial for delaying disc degeneration and promoting disc remodeling.

## Data Availability

The datasets used during the current study are available from the corresponding author on reasonable request.
